# Evolution of Site-Selection Stabilizes Population Dynamics, Promotes Even Distribution of Individuals, and Occasionally Causes Evolutionary Suicide

**DOI:** 10.1007/s11538-016-0198-5

**Published:** 2016-09-19

**Authors:** Kalle Parvinen, Åke Brännström

**Affiliations:** 1 Department of Mathematics and Statistics, University of Turku, 20014 Turku, Finland; 2 Department of Mathematics and Mathematical Statistics, Umeå University, 90187 Umeå, Sweden; 3 Evolution and Ecology Program, International Institute for Applied Systems Analysis, 2361 Laxenburg, Austria

**Keywords:** Adaptive dynamics, Evolution, First-principles derivation, Mechanistic modeling, Evolutionary suicide

## Abstract

Species that compete for access to or use of sites, such as parasitic mites attaching to honey bees or apple maggots laying eggs in fruits, can potentially increase their fitness by carefully selecting sites at which they face little or no competition. Here, we systematically investigate the evolution of site-selection strategies among animals competing for discrete sites. By developing and analyzing a mechanistic and population-dynamical model of site selection in which searching individuals encounter sites sequentially and can choose to accept or continue to search based on how many conspecifics are already there, we give a complete characterization of the different site-selection strategies that can evolve. We find that evolution of site-selection stabilizes population dynamics, promotes even distribution of individuals among sites, and occasionally causes evolutionary suicide. We also discuss the broader implications of our findings and propose how they can be reconciled with an earlier study (Nonaka et al. in J Theor Biol 317:96–104, 2013) that reported selection toward ever higher levels of aggregation among sites as a consequence of site-selection.

## Introduction

As you are reading this sentence, billions of organisms are engaged in a fierce struggle for the acquisition of territory and resources. While the population dynamics of these organisms are strictly dependent on multifarious factors such as individual behavior, resource availability, somatic growth, predation risk, mobility, diseases, and mate competition, important insights can often be attained by considering selected factors in isolation. The dynamics of populations that compete for access to or use of sites, such as mites, bean weevils, parasitic wasps, parasitic birds, aphids, and plants, are dependent on the distribution of individuals among sites and the competition within sites. These two factors are well-suited for mathematical description, and a modeling framework has been developed by Johansson and Sumpter (2003), Sumpter and Broomhead (2001), Brännström and Sumpter (2005b), and Anazawa (2009, (2010, (2012a, (2012b, (2014). The site-based framework developed in these papers allows for flexible and independent specification of the distribution of individuals among sites as well as the competitive interactions within sites.

One of the successes of the site-based framework was when Brännström and Sumpter (2005b) showed how combinations of assumptions on competition and clustering give rise to classical discrete-time population models. Allowing for two competing species, Anazawa (2014) further considered correlations in the number of individuals of each species within a site. While these two studies are forerunners in that they allow for other distributions than the Poisson distribution for individuals among sites (which results from random site choice with uniform probability), the distribution of individuals among sites is largely assumed without careful thought given to the underlying processes. The foremost of these processes is arguably evolution which will over time select for settlement strategies that offer individual advantages. One prior study by Nonaka et al. (2013) has considered how evolution affects site-selection in a site-based setting. That study, however, rests on the restrictive assumption that the desire to settle in an occupied site increases linearly with the number of individuals already there.

Here, we show for the first time how evolution of general site-selection strategies can be integrated with the site-based framework. To avoid introducing anthropogenic biases or artificially constraining the outcomes, we make only minimal assumptions on individual settlement and interaction and include the full feedback loop from individual behavior up to demographic change and from demographic composition down to selection on individual behaviors. To demonstrate the wide reach of the resulting eco-evolutionary system, we tackle three salient complementary questions which have until now not been systematically investigated.

First, we investigate how the evolution of site-selection strategies affect population dynamics and find that it generally stabilizes population dynamics independent of the type of interaction. Second, we study how the evolution of site-selection strategies affects the distribution of individuals among sites. We find that selection always favors a more even distribution of individuals among sites than what would result from a random site choice with uniform probability. This contrasts sharply with the conclusion by Nonaka et al. (2013) who found selection toward higher degrees of aggregation. Third, we consider the potential for evolutionary suicide and find that evolution of site-selection strategies occasionally causes the extinction of the population through either of two alternative routes.

We will establish and expand on the three conclusions above in Sect. 3, though we will first take the time to introduce and explain the mechanistic assumptions that underly them.

## Model and Methods

We consider the site-based framework of Sumpter and Broomhead (2001), Johansson and Sumpter (2003), and Brännström and Sumpter (2005b). The demographic dynamics unfold in non-overlapping generations. At each generation, individuals distribute themselves among resource sites according to their adopted behavioral strategy *s*, as described further below. The per-capita reproductive rate is then determined by the number of individuals at the same resource site. With the exception of rare mutations, the offspring faithfully inherit the behavioral strategies of their parents and emerge from the sites to constitute the next generation in a repetitive cycle. This gives the discrete-time dynamical system

1 $$\begin{aligned} x_{t+1}=f(x_{t})= \sum _{k=1}^{\infty }p_{k}(s,x_{t})k\varphi (k), \end{aligned}$$

in which $x_{t}$ is the population density in generation *t*, $p_{k}(s,x_{t})$ is the probability that a site contains *k* individuals and $\varphi (k)$ is the per-capita number of offspring of individuals in that site. In subsequent sections below, we describe how the probabilities $p_{k}$ emerge from individual behavior, the different interaction functions $\varphi (k)$ that we consider, and the resultant evolutionary dynamics.

### Individual Behavior and Resulting Population Dynamics

#### Distribution Among Sites

Let $\hat{x}$ denote the population density of individuals to be distributed among sites. We assume that individuals encounter sites randomly, according to the law of mass action with rate $\alpha$. At each encounter, the focal individual observes the number of individuals in the encountered site and has the option to settle in that site or to continue searching for other sites. In this work, we assume that once an individual has settled into a site, the decision is final, thus it cannot leave even if the site would later become crowded. In contrast, Parvinen et al. (2012) investigated the evolution of density-dependent emigration and immigration strategies in a metapopulation setting. The component $s_{k}$ of the vector-valued strategy of an individual is the probability to settle in an encountered patch containing *k* individuals at the moment. To keep the number of equations finite, we set a maximum number of individuals *K* that a site can contain, so that settlement into a site with *K* individuals is not possible. We choose *K* large enough so that this assumption only has negligible effects on our numerical results.

Let $\tau$ denote the time within the process of distributing individuals among sites. Let $x(\tau )$ denote the population density of individuals that at time $\tau$ are not yet distributed. Initially $x(0)=\hat{x}$. Let $p_{k}(\tau )$ denote the probability that a randomly chosen site has *k* individuals at time $\tau$. Initially, all sites are empty, thus $p_{0}(0)=1$ and $p_{k}(0)=0$ for all $k=1,2,\ldots ,K.$ For a monomorphic resident population, the assumptions made above mean that the probabilities $p_{k}(\tau )$ satisfy the differential equations

2 $$\begin{aligned} \frac{\mathrm {d}}{\mathrm {d}\tau }p_{0}(\tau )&=-\alpha x(\tau )s_{0}p_{0}(\tau )\nonumber \\ \frac{\mathrm {d}}{\mathrm {d}\tau }p_{k}(\tau )&=\alpha x(\tau )\left( s_{k-1}p_{k-1}(\tau )-s_{k}p_{k}(\tau )\right) \nonumber \\ \frac{\mathrm {d}}{\mathrm {d}\tau }p_{K}(\tau )&=\alpha x(\tau )s_{K-1}p_{K-1}(\tau ), \end{aligned}$$

and that the population density $x(\tau )$ of unsettled individuals satisfy

3 $$\begin{aligned} \frac{\mathrm {d}}{\mathrm {d}\tau }x(\tau )=-\alpha x(\tau )\sum _{k=0}^{K-1}s_{k}p_{k}(\tau ). \end{aligned}$$

For a polymorphic population with *n* morphs, the components $x(\tau )s_{k}$ need to be replaced by $\sum _{i=1}^{n}x_{i}(\tau )s_{k}^{i}$, in which $x_{i}(\tau )$ is the population density of non-distributed individuals with strategy $s^{i}$. Furthermore, let

4 $$\begin{aligned} y(\tau )=\sum _{k=0}^{K}kp_{k}(\tau ) \end{aligned}$$

denote the population density of settled individuals. Initially $y(0)=0$, and a straightforward calculation shows that

5 $$\begin{aligned} \frac{\mathrm {d}}{\mathrm {d}\tau }y(\tau )=\sum _{k=0}^{K}k\frac{\mathrm {d}}{\mathrm {d}\tau }p_{k}(\tau )=\alpha x(\tau )\sum _{k=0}^{K-1}s_{k}p_{k}(\tau )=-\frac{\mathrm {d}}{\mathrm {d}\tau }x(\tau ). \end{aligned}$$

In principle, we could assume that there is no restriction on the time allowed for site searching, and thus let all individuals to find a site. However, we find it more realistic that settlement into suitable sites is possible only during $0\leqslant \tau \leqslant T$. This means that too picky individuals (with low values of $s_{k}$) take the risk that they will not settle into a site at all, and thus fail to reproduce. The size-distribution of sites in the discrete-time population dynamics (1) is thus obtained by solving the differential Eqs. (2) and (5) until time *T*.

Note that the assumptions made above differ in several aspects from the earlier study by Nonaka et al. (2013), in which individuals were forced to choose one of the sites in a sequential but random order with different preferences based on the sizes of the sites. The assumptions made in the earlier model mean that at the moment of decision, the focal individual has perfect knowledge about the number of individuals in all sites, and the focal individual makes the decision into which site to settle based on that knowledge, and instantly settles into the chosen site. We find the present model, as described above, to be more realistic.

#### Interaction in Sites

After the individuals have selected their sites, they interact according to the interaction function $\varphi (k)$ giving the per-capita number of offspring as a function of the number of individuals *k* at the site. This function is meaningful only when $k=1,2,3,\ldots$. In this paper, we will consider scramble competition between individuals. This corresponds to situations in which reproductive output is greatly reduced whenever sufficiently many individuals occupy the same site (see Table 1). We focus on this competition type since the site-selection then becomes critical for the individual’s ability to reproduce.

In pure scramble competition, reproduction is possible only when an individual holds an entire site for itself, resulting in $\varphi (1)=b_1$ where $b_1$ is the number of offspring such an individual produces on average, while $\varphi (k)=0$ otherwise. We also consider a more general form of scramble competition in which two individuals sharing a site can also reproduce. While one might expect that, in such situations, $\varphi (1)>\varphi (2)$, we leave the specification of $\varphi (1)$ and $\varphi (2)$ arbitrary so as to include the possibility of mutualistic interactions. For these interaction functions, we also consider corresponding interaction functions with an Allee effect (Allee et al. 1949), where individuals require the presence of others to reproduce. Specifically, we assume that in the presence of an Allee effect, reproduction of lonely individuals is not possible.

**Table 1:** Investigated interactions and the corresponding nonzero values of the interaction function $\varphi (k)$

Competition	Without Allee effect, $\varphi (k)=$	With Allee effect, $\varphi (k)=$
Pure scramble	$b_1$ for $k=1$	$b_2$ for $k=2$
General scramble	$b_1$ for $k=1$, $b_2$ for $k=2$	$b_2$ for $k=2$, $b_3$ for $k=3$

#### Demographic Dynamics

If individuals always settle into a site they encounter, and *K* is infinite, the density of settled individuals at time *T* is $y(T)=\hat{x}(1-e^{-\alpha T})$, and the size-distribution of sites follows the Poisson distribution with mean *y*(*T*), thus $p_{k}(T)=e^{-y(T)}y(T)^{k}/k!$. If we let $T\rightarrow \infty$, and thus all individuals settle into a site, the resulting discrete-time dynamics with pure scramble competition results in

6 $$\begin{aligned} x_{t+1}=bx_{t}e^{-x_{t}}, \end{aligned}$$

which is the Ricker (1954) model. In the limit $T\rightarrow \infty$, we thus recover the classical site-based framework of Sumpter and Broomhead (2001) and Johansson and Sumpter (2003) who considered individuals distributed uniformly among sites (see also Royama 1992 for a closely related setting, as well as Appendix B of Brännström and Sumpter 2005b for a proof that the two frameworks are mathematically equivalent). For general strategy values, we are not able to give a simple analytical expression for the discrete-time population dynamics.

### Evolutionary Dynamics

#### Adaptive Dynamics

We investigate the evolutionary dynamics using techniques of adaptive dynamics (Dieckmann and Law 1996; Metz et al. 1996; Geritz et al. 1998 and others; see also the gentle introduction by Brännström et al. 2013). To facilitate the analysis, we assume that reproduction is clonal, and that the offspring are identical to their parents, except in rare instances when mutations occur. Since mutations are rare, we can assume that the resident population has reached a demographic attractor by the time the mutation occurs. The fate of the invading mutant is assessed from the mutant strain’s exponential growth rate while still rare in the resident environment. We will refer to this exponential growth rate as the invasion fitness (Metz et al. 1992).

Most studies on adaptive dynamics to date consider the evolution of a single trait value. By contrast, we consider the simultaneous evolution of several trait values which are jointly referred to as a vector-valued trait (see e.g., Brown et al. 2007; Parvinen et al. 2012). These traits are the probabilities $s_{k}$ that an individual assessing a site with *k* individuals will attach to that site. The principles behind adaptive dynamics of vector-valued traits are no different from those of a single trait value, but the conditions for assessing convergence stability (Leimar 2001, 2009) and evolutionary stability (Geritz et al. 2016) need to be extended to several dimensions.

For the model presented here, we find that there can be up to two attractors of the demographic dynamics. First, an extant population has a unique attractor which is a stable equilibrium, a periodic orbit, or a chaotic orbit. Second, with strong Allee effects, the extinction equilibrium is also a stable attractor. We next explain how the invasion fitness for a mutant with site-attachment probabilities different from those of the extant resident population is determined.

#### Invasion Fitness in the Present Model

Now, we study what happens for a mutant with strategy values $s_{k}^{\mathrm{mut}}$ in the site-distribution process. Since the mutant is globally rare, it will not affect the site-distribution process of the resident population. Therefore, the probability $p_{k}(\tau )$ that a randomly chosen site has *k* individuals at time $\tau$ is obtained by solving the differential Eqs. (2) and (3) for the resident population(s). Let $x^{\mathrm{mut}}(\tau )$ denote the probability that at time $\tau$, a randomly chosen mutant has not settled into any site. Furthermore, let $q_{k}^{\mathrm{mut}}(\tau )$ denote the probability that a randomly chosen mutant has settled into a site which contains *k* residents at time $\tau$. Note that the actual settlement time needs not to be exactly known, but it belongs to the interval $[0,\tau ]$. At time 0, no individuals have settled into sites, and thus $q_{k}^{\mathrm{mut}}(0)=0$ for all $k=0,1,2,\ldots ,K-1$. These probabilities satisfy the differential equations

7 $$\begin{aligned} \frac{\mathrm {d}}{\mathrm {d}\tau }q_{0}^{\mathrm{mut}}(\tau )&=\alpha x^{\mathrm{mut}}(\tau )s_{0}^{\mathrm{mut}}p_{0}(\tau )-\alpha x(\tau )s_{1}q_{0}^{\mathrm{mut}}(\tau )\nonumber \\ \frac{\mathrm {d}}{\mathrm {d}\tau }q_{k}^{\mathrm{mut}}(\tau )&=\alpha x^{\mathrm{mut}}(\tau )s_{k}^{\mathrm{mut}}p_{k}(\tau )+\alpha x(\tau )\left( s_{k}q_{k-1}^{\mathrm{mut}}(\tau )-s_{k+1}q_{k}^{\mathrm{mut}}(\tau )\right) \nonumber \\ \frac{\mathrm {d}}{\mathrm {d}\tau }q_{K-1}^{\mathrm{mut}}(\tau )&=\alpha x^{\mathrm{mut}}(\tau )s_{K-1}^{\mathrm{mut}}p_{K-1}(\tau )+\alpha x(\tau )s_{K-1}q_{K-2}^{\mathrm{mut}}(\tau ). \end{aligned}$$

The first term describes the rate at which encounters of a mutant and a site (without mutants) result in the settlement of that mutant into a site with currently *k* residents. Note that the mutant is rare, and thus the probability that a mutant encounters a site with at least one mutant is zero. The other terms describe the effect of a resident settling into a site which already contains one mutant and potentially some residents. Furthermore, we have

8 $$\begin{aligned} \frac{\mathrm {d}}{\mathrm {d}\tau }x^{\mathrm{mut}}(\tau )=-\alpha x^{\mathrm{mut}}(\tau )\sum _{k=0}^{K-1}s_{k}^{\mathrm{mut}}p_{k}(\tau ). \end{aligned}$$

Note that

9 $$\begin{aligned} \frac{\mathrm {d}}{\mathrm {d}\tau }\left( x^{\mathrm{mut}}(\tau )+\sum _{k=0}^{K-1}q_{k}^{\text {mut}}(\tau )\right) =0, \end{aligned}$$

which means that $x^{\mathrm{mut}}(\tau )+\sum _{k=0}^{K-1}q_{k}^{\text {mut}}(\tau )=1$ for all $\tau$. By solving (7) and (8) until time *T* we obtain the site-distribution of mutants. Since with probability one there can be only one mutant in a site, the expected number of mutant offspring per a mutant is

10 $$\begin{aligned} F(s^{\mathrm{mut}},{\mathbf {x}}_{\text {res}},{\mathbf {s}}_{\text {res}})=\sum _{k=0}^{K-1}q_{k}^{\mathrm{mut}}(s^{\mathrm{mut}},{\mathbf {x}}_{\text {res}},{\mathbf {s}}_{\text {res}})\varphi (k+1). \end{aligned}$$

In case the population-dynamical attractor of the resident is a fixed point ${\mathbf {x}}_{\text {res}}$, the basic reproduction number is directly given by (10). In general, if the attractor is an *n*-cyclic orbit consisting of points ${\mathbf {x}}_{\text {res}}^{j}$ for $j=1,\ldots ,n$, we have

11 $$\begin{aligned} R(s^{\mathrm{mut}})=\root n \of {\prod _{j=1}^{n}F(s^{\mathrm{mut}},{\mathbf {x}}_{\text {res}}^{j},{\mathbf {s}}_{\text {res}})}. \end{aligned}$$

The logarithm

12 $$\begin{aligned} r(s^{\mathrm{mut}})=\ln R(s^{\mathrm {mut}})=\frac{1}{n}\sum _{j=1}^{n}\ln F(s^{\mathrm{mut}},{\mathbf {x}}_{\text {res}}^{j},{\mathbf {s}}_{\text {res}}) \end{aligned}$$

is the invasion fitness of the mutant (Metz et al. 1992) and can be used in the evolutionary analyses of the model. A mutant may invade the resident only if $R(s^{\mathrm{mut}})>1$.

## Results

We allow site-selection to evolve under a range of plausible interaction functions, with and without an Allee effect. We first show in Sect. 3.1 that evolution of site-selection generally stabilizes the population dynamics. In Sect. 3.2, we show that it can surprisingly also lead to the extinction of the population by evolutionary suicide through either of two different routes. Finally, in Sect. 3.3, we demonstrate that the distributions of individuals that arise for evolutionarily stable site-selection strategies are underdispersed, i.e., non-aggregated.

### Population Dynamics are Generally Stabilized

Under pure scramble competition, the population dynamics are stabilized by evolution. This conclusion holds also in the presence of a strong Allee effect, though interestingly not always for interaction functions in between these two extremes.

#### Pure Scramble Competition

**Fig. 1:**
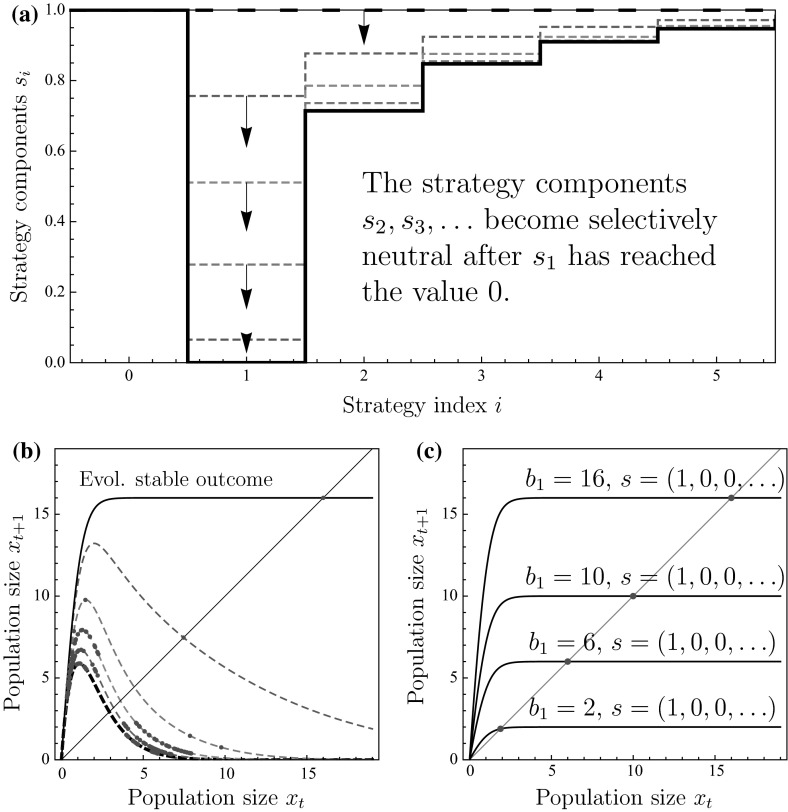
(Color figure online) Evolution stabilizes population dynamics under pure scramble competition: **a** a trait substitution sequence initiated with the always settle strategy $(1,1,\ldots ,1)$ (*thick dashed curve*) converging to strategy $(1,0,s_{2},s_{3},\ldots )$ (*thick curve*). **b** Discrete-time population models corresponding to the strategies in panel (**a**). **c** Discrete-time population models corresponding to the ESS strategy for different values of $b_{1}$. *Points* in panels **b** and **c** show the population-dynamical attractors. Parameters: Panel a and b: $b_{1}=16$. All panels: $\alpha T=2.5$

Under pure scramble competition, reproduction is possible only in sites with one individual and the interaction function consequently satisfies $\varphi (1)=b_{1}$ and $\varphi (k)=0$ for $k\ne 1$. Figure 1 shows how the evolution of site selection gradually leads from an initial state in which individuals always settle and frequently overexploit the available resource to a situation in which individuals only settle in empty sites (Fig. 1a). The individuals thus increasingly avoid overexploitation (Fig. 1b) and the evolutionary process eventually gives rise to a monotonically increasing return map (Fig. 1c) with corresponding stable population dynamics.

When the resident population has the always settle strategy and $K \rightarrow \infty$, the population size in sites is Poisson-distributed. The return map of the resident population is thus described by,

13 $$\begin{aligned} x_{t+1}=b_{1}p_{1}(T)=b_{1}\gamma x_{t}e^{-\gamma x_{t}}, \quad \text { where }\gamma =1-e^{-\alpha T}, \end{aligned}$$

which is the well-known Ricker model. This population exhibits period-doubling bifurcation to chaos as the parameter $b_{1}$ is increased (Fig. 1b, thick dashed curve). Under natural selection, the site-selection strategy gradually evolves to the state $s_{0}=1$, $s_{k}=0$ for $k=1,2,\ldots$, so that individuals eventually settle only into empty sites. In turn, the return map gradually changes from the Ricker model to a monotonically increasing curve (Fig. 1b). Once selection has come to a halt at the evolutionarily stable site-selection strategy, the resulting return map can be explicitly expressed as

14 $$\begin{aligned} x_{t+1}=f(x_{t})=b_{1}p_{1}(T)=b_{1}\frac{x_{t}\left( e^{\alpha T}-e^{\alpha Tx_{t}}\right) }{e^{\alpha T}-x_{t}e^{\alpha Tx_{t}}}. \end{aligned}$$

The extinction equilibrium $x=0$ is stable if $f'(0)=b_{1}\left( 1-e^{-\alpha T}\right) <1$. Further calculations show that the model (14) is increasing with respect to $x_{t}$ (the derivative $f'(x)>0$ for all $x\geqslant 0$), and $\lim _{x\rightarrow \infty }f(x)=b_{1}$. Furthermore, the function *f* is concave. This means that if $f'(0)<1$, the extinction equilibrium is globally stable, and if $f'(0)>1$, the extinction equilibrium is unstable, and there exists a unique positive equilibrium, and it is globally stable, even for large $b_{1}$ (Fig. 1c). Evolution of site-selection strategies thus stabilizes population dynamics at least under pure scramble competition.

Why does gradual evolution of site-selection result in individuals settling only into empty sites? Settling into a site occupied by one individual is not beneficial, because the reproductive potential vanishes. Settlement into sites with more individuals is not beneficial either. Actually, when the strategy $s_{1}$ has reached the value 0, the strategy components $s_{2},s_{3},\ldots$ become selectively neutral, because $s_{1}=0$ means that there will be no sites of size 2 and larger. When $s_{1}=0$, an individual who settles into an empty site will surely be able to reproduce. Therefore, $s_{0}$ should evolve to 1.

#### Pure Scramble Competition with an Allee Effect

**Fig. 2:**
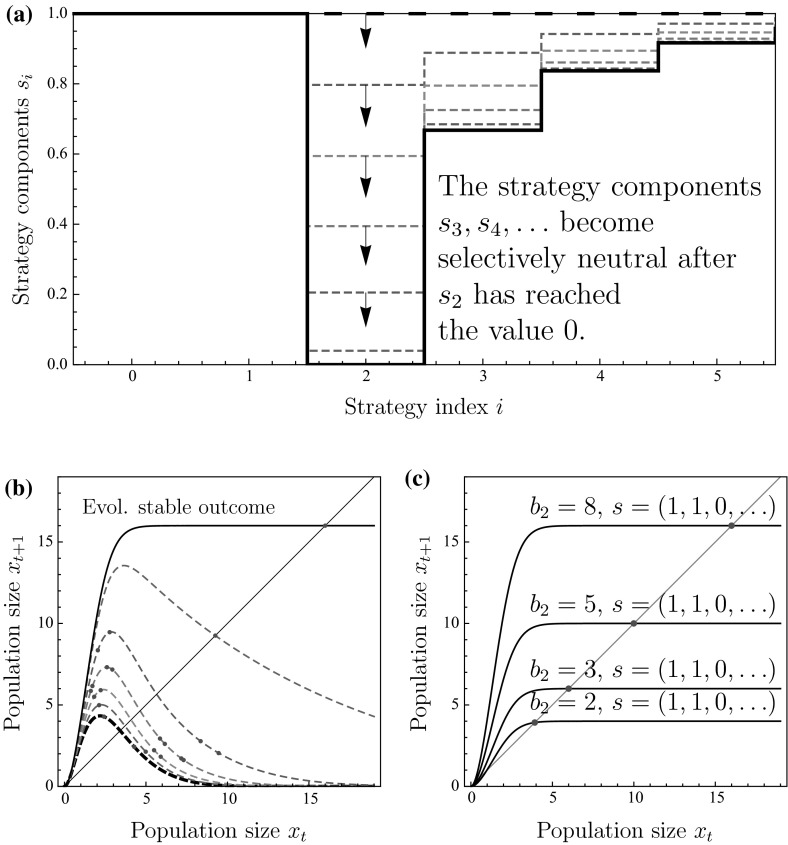
(Color figure online) Evolution stabilizes population dynamics also under pure scramble competition with Allee effect: **a** a trait substitution sequence initiated with the always settle strategy $(1,1,\ldots ,1)$ (*thick dashed curve*) converging to strategy $(1,1,0,s_{3},s_{4},\ldots )$ (*thick curve*). **b** Discrete-time population models corresponding to the strategies in panel (**a**). **c** Discrete-time population models corresponding to the ESS strategy for different values of $b_{2}$. *Points* in panels **b** and **c** show the population-dynamical attractors. Parameters: Panel a and b: $b_{2}=8$. All panels: $\alpha T=2.5$

Next, we consider the interaction function with $\varphi (2)=b_{2}$ and $\varphi (k)=0$ for $k\ne 2$. This corresponds to pure scramble competition with a strong Allee effect, in which exactly two individuals are required in a site to successfully reproduce. Figure 2a illustrates how site-selection will now evolve to the strategy of settling whenever a site contain either none or exactly one individual. While this affects the precise from of the return map, the qualitative findings remain the same as for pure scramble competition. Again, we have Poisson-distributed population sizes in sites when the resident individuals always settle into encountered sites. The population dynamics satisfy

15 $$\begin{aligned} x_{t+1}=b_{2}p_{2}(T)=b_{2}\gamma ^{2}x_{t}^{2}e^{-\gamma x_{t}}, \quad \text { where }\gamma =1-e^{-\alpha T}. \end{aligned}$$

For small $b_{2}$, the population is not viable. When $b_{2}$ is increased, two positive equilibria appear, of which the larger one is stable. Increasing $b_{2}$ further will cause the larger positive equilibrium to become unstable, and we observe a cascade of period-doubling bifurcations leading to chaos. Eventually, the chaotic attractor will collide with the lower unstable positive equilibrium, beyond which the population is not viable due to the Allee effect. For a similar series of bifurcations with respect to a fecundity parameter, see Fig. 12 of Parvinen (2005) and Fig. 3c of Parvinen and Dieckmann (2013). Under natural selection, the site-selection strategy gradually evolves to the state $s_{0}=1$, $s_{1}=1,$ and $s_{k}=0,$ for $k=1,2,\ldots$, so that individuals eventually settle only into sites that are either empty or contain exactly one individual. In turn, the return map gradually changes from the Ricker model to a monotonically increasing curve (Fig. 2b). The evolutionary process eventually results in the monotonically increasing return map shown as the thick solid curve in Fig. 2b (see also Fig. 2c for examples of how the resulting return map changes depending on individual fecundity $b_{2}$) with corresponding stable population dynamics.

Why does the settlement strategy evolve to a strategy of settling when the site contains either none or exactly one individual? If an individual encounters a patch with two or more individuals, settlement into such a site would mean that none of them can reproduce. Therefore, the selection gradient for each strategy component $s_{2}$, $s_{3},\ldots$ is negative (unless some $s_{k}=0$ making $s_{k+1}$, $s_{k+2},\ldots$ selectively neutral). Consider now the case $s_{2}=0$: if an individual encounters a site with 1 individual, it can guarantee its reproductive success by settling into that site. Therefore, $s_{1}$ should evolve to 1. How should the strategy component $s_{0}$ evolve when $s_{1}=1$ and $s_{2}=0$? If the population density is large and an individual settles into an empty site, it is very likely that another individual will settle there too, resulting in reproductive success. In contrast, if the population density is low, an individual settling into an empty site may encounter the risk that nobody will manage to settle there as well, and it could instead be better to search for a site which already contains one individual. We investigated this possibility numerically and observed that there is a threshold population density so that for smaller population densities, values smaller than 1 for the strategy component $s_{0}$ would be beneficial. However, the population density in a stable positive equilibrium appears to always be larger than the aforementioned threshold. Therefore, the strategy $s_{0}=s_{1}=1$, $s_{k}=0$ for all $k=2,3,\ldots$ is an evolutionary attractor and uninvadable (evolutionarily stable).

#### General Scramble Competition

We now investigate the evolution of site-selection under general scramble competition through the interaction function,

16 $$\begin{aligned} \varphi (k)=\left\{ \begin{array}{ll} b_{1}, &{} \quad \text {for }k=1,\\ b_{2}, &{} \quad \text {for }k=2,\\ 0, &{} \quad \text {otherwise}. \end{array}\right. \end{aligned}$$

**Fig. 3:**
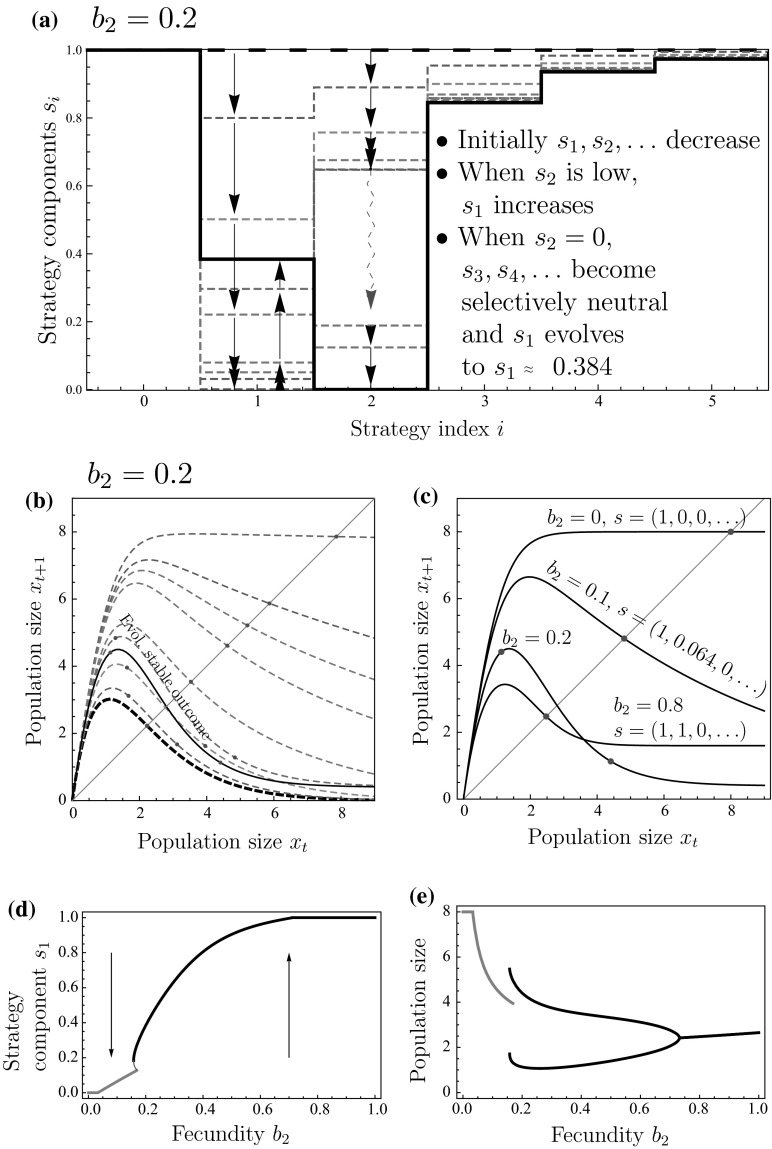
(Color figure online) Evolution can result in cyclic population dynamics under general scramble competition: **a** a trait substitution sequence initiated with the always settle strategy $(1,1,\ldots ,1)$ (*thick dashed curve*) converging to strategy $(1,s_{1},0,s_{3},s_{4},\ldots )$ with $s_{1}\approx 0.384$ (*thick curve*). **b** Discrete-time population models corresponding to the strategies in panel a. **c** Discrete-time population models corresponding to the ESS strategy for different values of $b_{2}$. *Points* in panels **b** and **c** show the population-dynamical attractors. **d** Evolution of the strategy component $s_2$ as a function of fecundity $b_2$. *Curves* in panel d show evolutionarily singular strategies. **e** Population-dynamical attractors corresponding to the evolutionarily attracting singular strategies illustrated in panel d. Parameters: Panel a and b: $b_{2}=0.2$. All panels: $\alpha T=2.5$, $b_{1}=8$

As this interaction function is intermediate between pure scramble competition and pure scramble competition with Allee effect, which have been explored in the two previous sections, one might hypothesize that the evolved site-selection strategy and resulting population dynamics will also be intermediate between the two. For most values of $b_{1}$ and $b_{2}$, this intuition bears out. Evolution will result in a site-selection strategy identical to that of pure scramble or pure scramble with Allee effect, and the return map will be monotonically increasing with stable population dynamics. Surprisingly, other outcomes are possible when it is much advantageous for individuals to hold a site on their own rather than share it with a conspecific, i.e., when $b_{2}\ll b_{1}$, Fig. 3a illustrates the evolution of site selection in a representative case. Starting from the strategy of always settling, the selection gradient for each strategy component $s_{1}$, $s_{2},\ldots$ is initially negative with the rate of evolutionary change being highest for strategy component $s_{1}$. As soon as $s_{1}=0$ has been fixated in the population, selection for the remaining strategy components $s_{2}$, $s_{3},\ldots$ becomes neutral and random drift dominates. Random drift will at some point cause the probability of settling into sites already containing two individuals to become small. Suddenly, it is advantageous for individuals to occasionally forego an opportunity to settle into a site already containing one individual. Selection for $s_{1}$ becomes positive while selection for $s_{2}$ ceases to be neutral and again becomes negative. This can result in such a settlement strategy that individuals always settle in empty sites, sometimes settle in sites containing exactly one individual, and never settle into sites with two or more individuals. While the emerging settlement strategy is novel, it remains intermediate between pure scramble competition and pure scramble competition with Allee effect. Does this mean that the return map will also be intermediate between the two and hence again monotonically increasing?

**Fig. 4:**
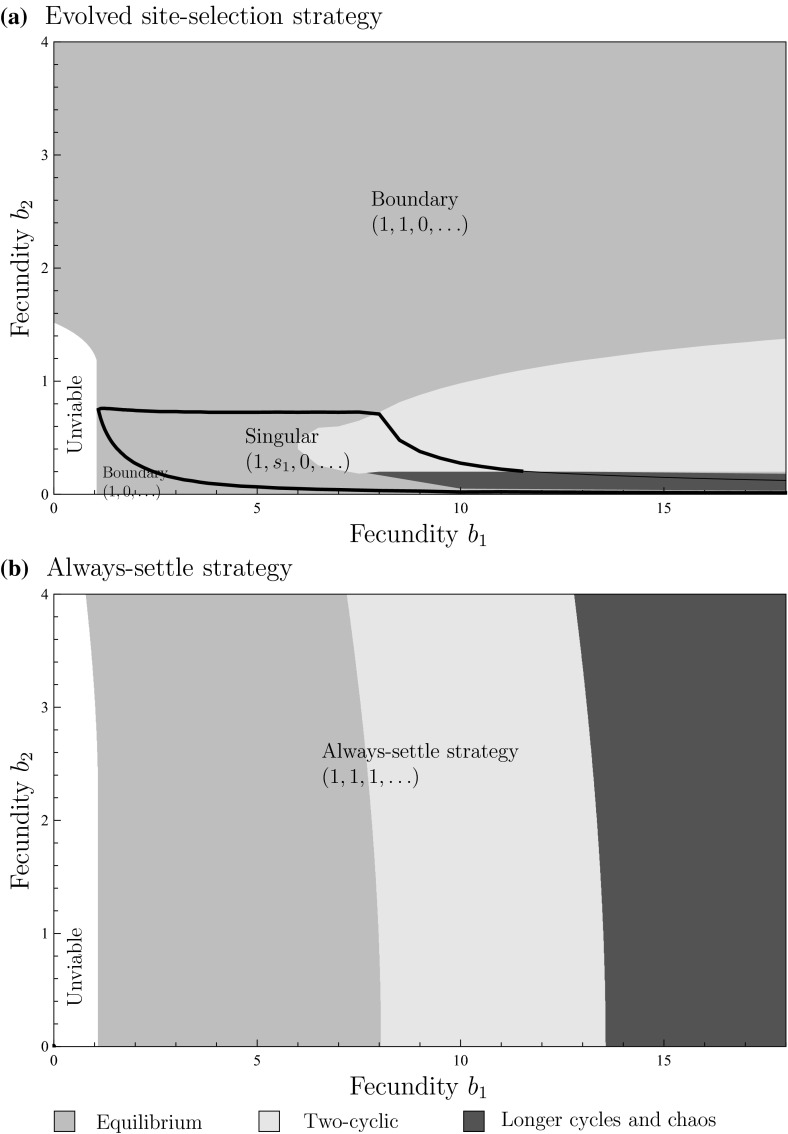
(Color figure online) Evolution typically stabilizes population dynamics under general scramble competition: **a** Evolved settlement strategy and the corresponding population dynamics for different combinations of fecundities $b_{1}$ and $b_{2}$. The evolved strategy is either of the boundary strategies $(1,0,\ldots )$ and $(1,1,0,\ldots )$, or a singular strategy $(1,s_{1},0,\ldots )$ with $0<s_{1}<1$. **b** Type of the population-dynamical attractor for the always settle strategy $(1,1,1,\ldots )$. *Shading* illustrates different types of population dynamics

Figure 3b shows that evolution initially causes the return map to approach a monotonic curve, and hence increase population-dynamical stability. However, when the evolutionary process continues after some time of random drift, the trend is reversed and the population increasingly experiences overcompensation, eventually resulting in the return map corresponding to the solid curve in Fig. 3b with associated periodic population dynamics. The generality of this finding is explored in Fig. 3c–e, and the conclusion is that non-equilibrium dynamics as a result of evolution of site-selection is possible only in a limited range of the parameter $b_{2}$. Figure 3c shows return maps for different values of $b_{2}$ with the corresponding population-dynamical attractor shown in Fig. 3e. It is only for approximately $0.2\lessapprox b_{2}\lessapprox 0.8$ that the return map does not give rise to stable population dynamics. To get a better view of the generality, the type of the ESS strategy and the type of the population-dynamical attractor with that strategy are shown with respect to the parameters $b_{1}$ and $b_{2}$ in Fig. 4a. Figure 4a shows that for most of the parameter values evolution results in the strategy $(1, 1, 0, 0, \ldots )$ according to which individuals always settle to empty sites or sites with one individual, and never to sites with two or more individuals. Furthermore, in contrast to the type of population dynamics with the always settle strategy (Fig. 4b), evolution mostly results in equilibrium population dynamics. Thus, although general scramble competition offers an interesting twist by occasionally allowing novel settlement strategies and non-equilibrium population dynamics, we again find that evolution of site-selection generally stabilizes population dynamics.

### Evolutionary Suicide Through Two Different Routes

Unexpectedly, while the evolution of site-selection generally tends to stabilize population dynamics, it can cause the extinction of the population through evolutionary suicide (Ferrière 2000; Gyllenberg and Parvinen 2001; Gyllenberg et al. 2002; Parvinen 2005; Parvinen and Dieckmann 2013). In Sect. 3.2.1, we show that this can happen when the population is subject to pure scramble competition with strong Allee effect. The importance of this finding is underscored in Sect. 3.2.2 where we show that the range of conditions under which evolutionary suicide unfolds increases for more general interaction functions with strong Allee effects.

**Fig. 5:**
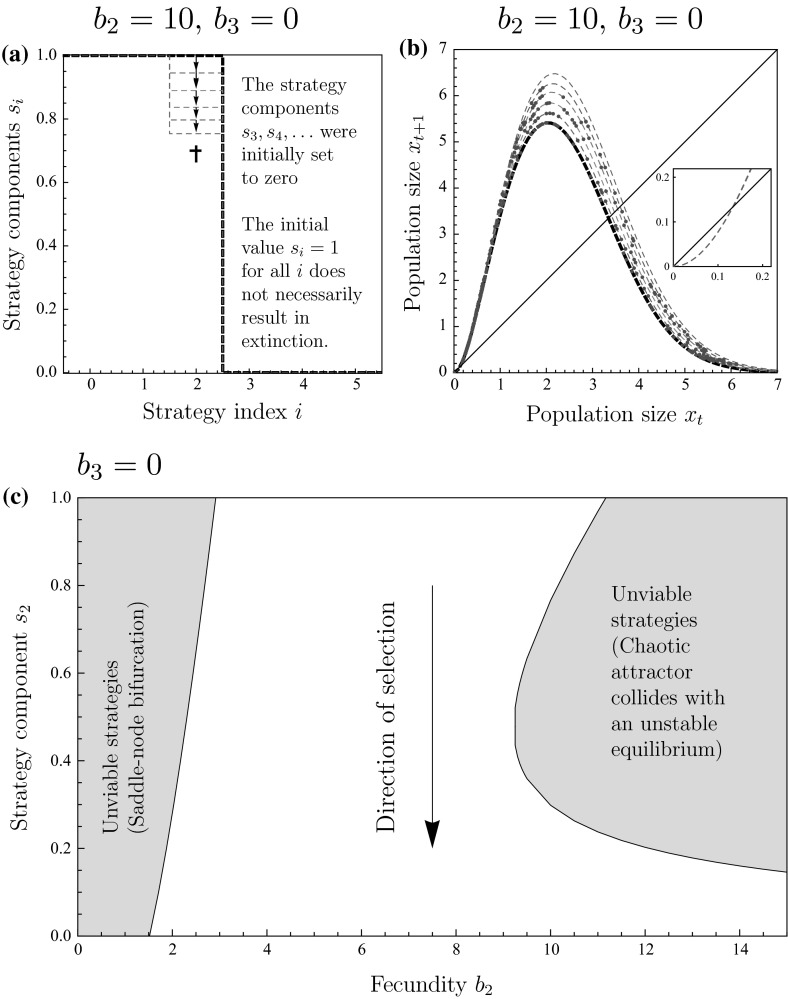
(Color figure online) First route to evolutionary suicide: **a** a trait substitution sequence initiated with the strategy $s=(1,1,1,0,\ldots )$. The strategy component $s_2$ decreases until the population goes extinct. **b** Explanation of the extinction. As a consequence of the decrease in $s_2$, the return map for the population dynamics changes so that a chaotic attractor collides with an unstable equilibrium, beyond which the population is no longer viable. **c** Phase diagram illustrating when evolution of strategy component $s_2$ results in collision with an unstable equilibrium and eventual extinction. The interaction function is given by Eq. 17

#### Extinction Through Changes in Chaotic Attractor

In the story thus far, we have emphasized the potential of evolution of site-selection to stabilize population dynamics. Under very special circumstances, the evolutionary process may result in the extinction of the population through either of two routes. The first of these can arise under pure scramble competition with strong Allee effect, provided that the fecundity parameter $b_{2}$ is large enough for chaotic population dynamics. Figure 5a–c illustrates one representative case. Starting from a strategy of settling when a site contains two individuals or less, $s=(1,1,1,0,\ldots )$, population dynamics is chaotic, and selection will reduce the strategy component $s_{2}$ as individuals in sites with three or more individuals will produce no offspring at all (Fig. 5a). This causes the return map to change (Fig. 5b) and consequently affects the chaotic attractor. For a range of combinations of $b_{2}$ and $s_{2}$, the population is unviable (Fig. 5c). The change from a viable chaotic attractor to unviability happens when the population size first reaches the peak of the return map, but then fecundity is so small because of overcompensation, that the population size in next generation falls below the Allee threshold (inset of Fig. 5b). As $s_{2}$ decreases through gradual evolution, the chaotic attractor eventually changes to allow population sizes below the Allee threshold. From this low abundance, the population cannot recover and extinction is an inevitable outcome.

#### Extinction Through Intraspecific Competition

A qualitatively different type of evolutionary suicide can be revealed if we broaden our investigation to more general interaction functions with strong Allee effects. Specifically, we will consider the interaction function

17 $$\begin{aligned} \varphi (k)=\left\{ \begin{array}{ll} b_{2}, &{} \quad \text {for }k=2,\\ b_{3}, &{} \quad \text {for }k=3,\\ 0, &{} \quad \text {otherwise}. \end{array}\right. \end{aligned}$$

Similarly to general scramble competition, general scramble competition with Allee effect occasionally introduces a conflict between the interests of the individual and those of the population. This roughly occurs when the total number of individuals emerging from a site with three individuals is less than the number emerging from a site with two individuals, i.e., when $3b_{3}<2b_{2}$. In such cases, an individual encountering a site with two individuals might find it advantageous to settle with high chances of securing $b_{3}$ offspring rather than to keep searching for a more promising site in what might ultimately prove to be a vain effort. The population, however, would be better served if the individual did continue searching in the hope of colonizing and exploiting more sites. In certain cases, the negative consequences of individual self-interest may be so large that they induce extinction.

**Fig. 6:**
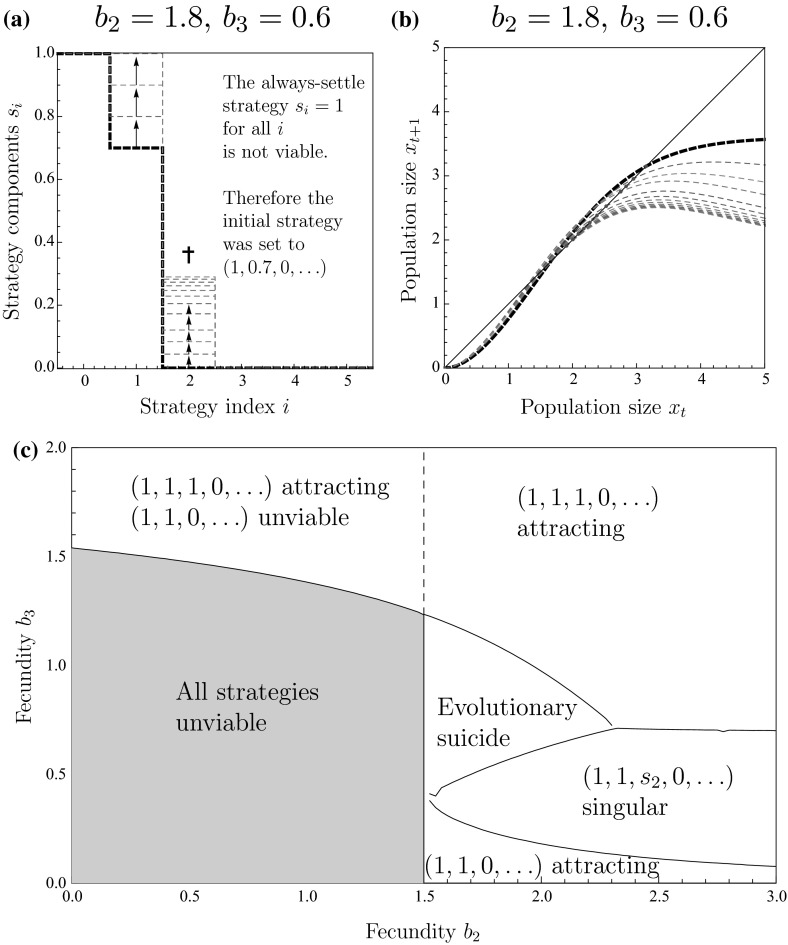
(Color figure online) Second route to evolutionary suicide: **a** a trait substitution sequence results in the strategy $s=(1,1,s_3,0,\ldots )$ becoming fixated in the population. **b** As a consequence, the return map for the population dynamics changes and the stable equilibrium vanishes in a saddle-node bifurcation. This results in the extinction of the population. **c** Phase diagram illustrating the resulting evolutionary outcomes for different values of $b_2$ and $b_3$. The interaction function is given by Eq. 17

**Fig. 7:**
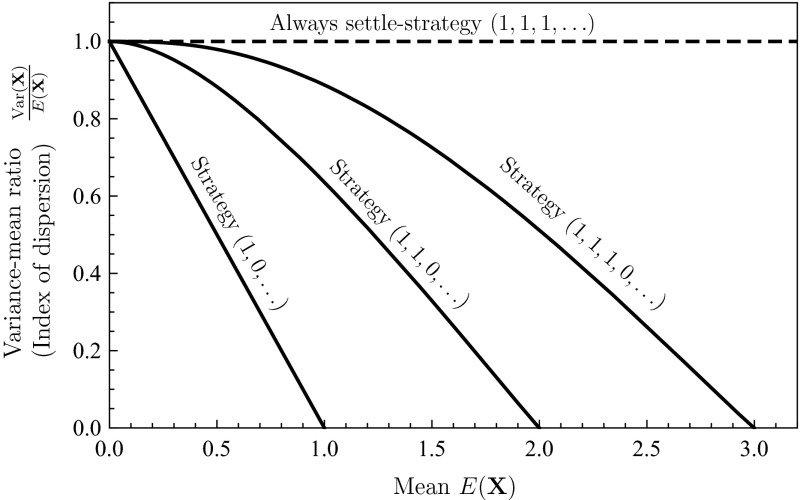
Evolution promotes even distribution of individuals: dependence of the mean and the variance-mean ratio (the dispersion index) for the distribution of individuals among sites for four site-selection strategies. The always settle strategy results in the Poisson distribution, for which the variance-mean ratio is always one. The other illustrated strategies are of form given by (18), which is the form of evolutionary stable site-selection strategies that the interaction functions we have considered have all given rise to. These site-selection strategies always result in underdispersed (non-aggregated) distributions, for which the variance-mean ratio is less than one

Figure 6a–c shows how evolution of site-selection may induce evolutionary suicide. Starting from such a strategy that individuals always settle in empty sites and sometimes in sites with one individual, selection is positive for both strategy components $s_{1}$ and $s_{2}$. The strategy component $s_{1}$ will reach the value 1, and the strategy component $s_{2}$ will increase further. As $s_{2}$ increases, more and more sites will be occupied by three individuals. This confers an individual advantage, but the competition with conspecifics for resources depresses the realized fecundity (since $3b_{3}<2b_{2}$). Fig. 6b shows how the return map is gradually depressed and eventually falls below the diagonal line $x_{t+1}=x_{t}$. At this point, the population size decreases with every iteration, eventually resulting in the extinction of the population.

### Distribution of Individuals Among Sites Becomes More Uniform

As we have seen in the previous sections, evolution of site selection strategies generally stabilizes population dynamics and, somewhat counterintuitively, occasionally results in the extinction of the population through evolutionary suicide. Figure 7 shows that the evolution of site-selection makes the observed distribution of individuals among sites more uniform (underdispersed) compared with the strategy in which individuals always settle in a site they encounter. If we change parameters in such a way that the evolved strategy does not change, but the average number of individuals in a site increases, we observe that the distribution becomes more and more underdispersed, until all sites are occupied with the same amount of individuals, resulting in maximal uniformity. If two different strategies result in the same average number of individuals in a site, the strategy to settle only into empty sites results in the most uniform (underdispersed) distribution. Such strategy is, for example, always reached through evolution of site-selection under pure scramble competition. As the interaction function changes to allow for reproductive output also with two or more individuals at the site, the distribution typically becomes less uniform but still more so than a completely random site choice (the always settle strategy).

To substantiate the aforementioned conclusions, we assess the index of dispersion of the resulting distributions of individuals per site by considering their variance-to-mean ratio, roughly the spread in the number of individuals per site divided with the average number of individuals per site. The always settle strategy, corresponding to a Poisson distribution when *K* is large, has a variance-to-mean ratio of exactly one. Distributions with lower variance-to-mean ratios are more uniform (underdispersed) and those with higher variance-to-mean ratios more aggregated (overdispersed). The interaction functions we have considered have all given rise to evolutionary stable site-selection strategies of the form,

18 $$\begin{aligned} {(s_{0},s_{1},\ldots )}\quad \text {with}\quad s_{k}=\left\{ \begin{array}{ll} 1, &{} \quad k\leqslant \left\lfloor z\right\rfloor , \\ z-\left\lfloor z\right\rfloor , &{}\quad \left\lfloor z\right\rfloor <k\leqslant \left\lceil z\right\rceil , \\ 0, &{}\quad k>\left\lceil z\right\rceil . \end{array}\right. \end{aligned}$$

An individual with this strategy will always settle if the site contains less than *z* individuals and never settle if it contains more than $z+1$ individuals. For non-integer values of *z*, there is an intermediate case in which the individual sometimes settles. Our investigations indicate that the value of *z* depends on how fast the per-capita reproduction rate declines with the number of individuals, with the lowest value $z=1$ corresponding to pure scramble competition. The site-selection strategies of form (18) give rise to a “truncated” Poisson distribution. Especially, for integer values of *z*, the probability $p_{k}$ of having exactly *k* individuals in a site is given by

19 $$\begin{aligned} p_{k}=\left\{ \begin{array}{ll} \frac{\lambda ^{k}}{k!}e^{-\lambda }, &{}\quad k\leqslant z,\\ \sum _{j=z+1}^{\infty }\frac{\lambda ^{j}}{j!}e^{-\lambda }, &{} \quad k=z+1,\\ 0, &{} \quad k>z+1. \end{array}\right. \end{aligned}$$

Figure 7 shows how the variance-to-mean ratio depends on the average numbers of individuals per site for the three distributions corresponding to $z=1$, $z=2$ and $z=3$. The strategy of always settling, corresponding to a Poisson distribution when the number of sites is large, is also shown for comparison. All three distributions are more uniform than the Poisson distribution and the index of dispersion decreases (uniformity increases) when the average number of individuals per site increases and when the value of *z* decreases. Note that the mean of the distribution is not a parameter that can be freely chosen, but instead emerges from the chosen interaction function and the evolved strategy.

## Discussion

Site-based models have a long history in theoretical ecology where they have been used to elucidate how the population dynamics are affected by competition for resources. These studies have generally imposed assumptions on how individuals choose which site to utilize. The most common assumption is that individuals choose sites entirely at random, with uniform probability, although aggregated distributions (Brännström and Sumpter 2005b) and distributions arising from spatial structure (Brännström and Sumpter 2005a) have also been considered. Rather than assuming a distribution a priori, we have allowed site-selection strategies to evolve freely without any constraints imposed. We found that, as a consequence, the population dynamics are typically stabilized and the resulting distribution of individuals more uniform compared to Poisson distribution resulting from the canonically assumed entirely random site choice (corresponding to the always settle strategy). Interestingly, we also found that the evolution of site-selection strategies occasionally results in evolutionary suicide.

*Why do we not see individuals aggregating in sites?* Our study can be compared with, and was to a large extent motivated by, a recent publication by Nonaka et al. (2013) which investigated whether the presence of an Allee effect can promote the evolution of preferential attachment in a related site-based setting. That study concluded that aggregation tendencies either did not evolve, or resulted in runaway evolution leading to evolutionary suicide. The site-selection strategies in that study were restricted to a single trait value representing the increased propensity of an individual to attach to an already populated site. By contrast, we considered a vector-valued trait allowing full freedom in the evolved site-selection strategies. An individual might, for example, have the strategy of avoiding empty sites, preferring to settle in sites containing few individuals, and entirely avoiding overcrowded sites. That we allow for any plausible site-selection strategy is most likely the reason why we do not find evolution of preferential attachment. Instead, the evolutionarily stable site-selection strategies that emerge are characterized by avoidance of overcrowded sites and thus result in more uniform distributions of individuals among sites than a fully random site choice (corresponding to the always settle strategy). The potential for evolutionary suicide remains, though, and this scenario can unfold through either of two possible routes. There is also a difference in the interaction functions considered. While we have systematically explored interaction functions for scramble competition under the assumption that reproduction is never possible with four or more individuals at a site, Nonaka et al. (2013) considered a version of Royama (1992)’s scramble interaction function in which reproductive output declines geometrically with the number of individuals at the site which had been extended to incorporate an Allee effect. We do not believe that this difference is fundamental, though further work is needed to investigate the evolution of site-selection strategies for more general interaction functions.

*Evolution and population-dynamical stability* While our study is the first to investigate unconstrained evolution of site-selection strategies in the site-based framework, other authors have considered how evolution of salient individual traits affect the population dynamics. The general conclusion emerging from these studies is that evolution often but now always stabilizes population dynamics. This message is reinforced by theoretical studies which using stylized demographic models, consider evolution of traits affecting population dynamics, such as traits related to carrying capacity, intrinsic growth rate, maturation rate, and mortality rate (e.g., Gatto 1993; Doebeli and Koella 1995; Ebenman et al. 1996). Similar results have also been obtained at the community level, with Zeineddine and Jansen (2005) considering two competing strains of *Drosphilia* and Loeuille (2010) investigating a two-species Lotka–Volterra system under different species interactions. The conclusions of the aforementioned studies should be contrasted with Ferrière and Gatto (1993) who considered an age-structured model with a trade-off between adult survival and recruitment rate, and Johst et al. (1999) who found that in a spatially-structured model, evolution of dispersal rate and a trait determining dynamical complexity can result in complex population dynamics. Moreover, Mellard and Ballantyne (2014) found that the stability of an evolved community is generally less than the highest attainable; we do not find this very surprising however and feel that the question should rather be whether the evolved community is more stable than a randomly assembled extant community. The question whether evolution stabilizes population dynamics has also been studied empirically. Mueller et al. (2000) found that while important life-history characteristics of *Drosphilia* did evolve, there were no noticeable effects on population-dynamical stability. A more recent study by Prasad et al. (2003), also on *Drosphilia*, did, however, detect stabilizing effects of evolution on the population dynamics. Although there is no obvious reason to expect that the selfish interests of individuals should include stabilization of population and community dynamics, the latter is apparently often an outcome of evolutionary processes.

*Ovipositioning strategies of insects* A recurrent theme in the literature over the last decades has been the dilemma faced by ovipositioning insects, and in particular parasitic insects. In these studies, females are faced with the problem of distributing several eggs over discrete patches (such as susceptible hosts) which are encountered sequentially. Among salient examples, Mangel (1987) used a process-based model to explain why the actual clutch size laid by a female parasite in an encounter is often lower than the clutch size found to be optimal in experiments. Mangel (1989) applied similar methods to understand an experiment by Collins and Dixon (1986) on the parasitization of Sycamore aphids by *M. pseudoplatani*. Horng (1994) considered an optimization problem for bean weevils who sequentially encounter beans with variable number of eggs each and have to decide whether to oviposit or forego the opportunity in the hope of a better opportunity before the terminal time. Using a framework somewhat similar to ours, Ives (1989) studied how the ESS clutch size of insects depend on type of competition among larvae within discrete patches. One study on host acceptance by parasitic wasps by Plantegenest et al. (2004) has also shown that stochastic (random) strategies can sometimes be optimal, analogous to the singular probabilistic strategies that we found. Our study complements this body of work by explicitly considering effects on and by inter-generational population dynamics.

*Evolution of sociality* Several authors have used an eco-evolutionary approach to explore the evolution of sociality. Avilés (1999) considered how two opposing forces, cooperative interactions and negative density-dependence, results in the dynamics of social groups (effectively analyzing the Ricker model with an Allee effect and related models). Aviles et al. (2002) extended this framework to include the evolution of grouping tendencies, in which the probability to join a group was assumed proportional to the product of (i) the individual’s grouping tendency, (ii) the average grouping tendency of individuals in the group, and (iii) a linearly decreasing function of group size which equaled zero when the per-capita number of offspring in the group was less than one. More recent work on the evolution of sociality include Purcell et al. (2012), Shen et al. (2014), and Van Veelen et al. (2010). Using the methods presented here to advance understanding of the evolution of sociality would be an interesting extension, but it is far from being the only promising one.

*Promising extensions * Several extensions can be conceived. First, we could consider the option of depositing a selective number of eggs at a site while retaining the remaining eggs for possible future encounters. Second, the decision of whether to deposit eggs could depend on the number of eggs that the individual still retains and the time in the season. This would likely make the individual more selective for the last few remaining eggs and less selective as the time passes. Third, if we interpret site-selection not as the oviposition of eggs but as the habitat choice of individuals, we can include factors such as territorial defense and local war of attrition in which an individual may choose to leave a patch on which it has already settled when conditions become unfavorable. Fourth, in line with the literature on the evolution of sociality, it would be possible to consider strategies where existing group members have a say in the matter, for example by not allowing more members to join a group. Fifth, in our study we have systematically explored interaction functions corresponding to scramble competition with and without Allee effects, in which the reproductive output is identically zero whenever four or more individuals settle in a patch. It would be interesting to consider a more general class of interaction functions including, in particular, contest competition. This would likely change the conclusions presented here, as there is always some benefit of settling in contest competition even if the site is already fairly crowded. Finally, after imposing individual variability in competitive ability or other factors, it would be very interesting to study how the evolved strategies depend on these factors. For example, we expect that strong competitors will adopt aggressive strategies of settling fairly soon even in crowded sites while individuals with lesser competitive ability might search longer for empty patches.

## Conclusions

Population dynamics arise from the life-history, interactions, and movement of individuals. Here, we have contributed to a better understanding of individual movement by considering the evolution of site-selection strategies for species reproducing in discrete resource sites. In addition to characterizing evolutionarily stable site-selection strategies, our work reveals that evolution of site-selection stabilizes population dynamics, promotes even distribution of individuals, and occasionally causes the extinction of the entire population through evolutionary suicide. Continuing our work in the direction of an integrative and synthetic framework for site-selection and group formation is an exciting and important challenge for the future.
